# Readiness planning: how to go beyond “buy-in” to achieve curricular success and front-line performance

**DOI:** 10.1186/s41077-024-00317-z

**Published:** 2024-12-23

**Authors:** Christopher J. Roussin, Grace Ng, Mary K. Fey, James A. Lipshaw, Henrique P. Arantes, Jenny W. Rudolph

**Affiliations:** 1 Applied Learning for Performance and Safety (ALPS), Center for Medical Simulation, 100 First Avenue, Suite 400, Boston, MA 02129 USA; 2 Harvard Medical School, Boston, USA; 3 Department of Anesthesia Critical Care and Pain Medicine, Center for Medical Simulation, Beth Israel Deaconess Medical Center, 100 First Avenue, Suite 400, Boston, MA 02129 USA; 4 Faculty Development Programs, Center for Medical Simulation, 100 First Avenue, Suite 400, Boston, MA 02129 USA; 5 Instructional Design & Media, Center for Medical Simulation, 100 First Avenue, Suite 400, Boston, MA 02129 USA; 6 IMEPAC, 1889 Minas Gerais Street, Araguari, Minas Gerais Brazil; 7 Hospital Universitario Sagrada Familia (HUSF), 1889 Minas Gerais Street, Araguari, Minas Gerais Brazil; 8 Innovation, Center for Medical Simulation, 100 First Avenue, Suite 400, Boston, MA 02129 USA

**Keywords:** Simulation, Curriculum design, Change leadership, Training design, Healthcare quality, Patient safety, Clinical outcomes

## Abstract

Simulation program staff and leadership often struggle to partner with front-line healthcare workers, their managers, and health system leaders. Simulation-based learning programs are too often seen as burdensome add-ons rather than essential mechanisms supporting clinical workforce readiness. Healthcare system leaders grappling with declining morale, economic pressure, and too few qualified staff often don’t see how simulation can help them, and we simulation program leaders can’t seem to bridge this gap. Without clear guidance from front-line clinicians and leaders, the challenge of building and maintaining sustainably relevant simulation offerings can seem overwhelming. We argue that three blind spots have limited our ability to see the path to collaborations that support front-line workforce readiness: We wrongly assume that our rigor in designing and delivering programs will lead to front-line participant engagement and positive impact, we overestimate the existence of shared priorities, mindsets, and expertise with our would-be partners, and we contribute to building a façade of superficial education compliance that distracts from vital skill development. How do we design simulation-based training programs that are valued, supported, and sustained by key partners over time? (1) By seeing ourselves as partners first and designers second; (2) by using a boundary spanning design process that shifts the primary psychological ownership of training outcomes to our partners; and (3) by focusing this shared design process on workforce readiness for the situations that our healthcare partners care about most. Drawing on lessons from more than 800 readiness plans developed by participants in our courses and the authors’ successes and mistakes in partnering with healthcare teams for front-line readiness, we introduce the concepts, commitments, and practices of “readiness planning” along with three detailed examples of readiness planning in action.

**Graphical Abstract:**

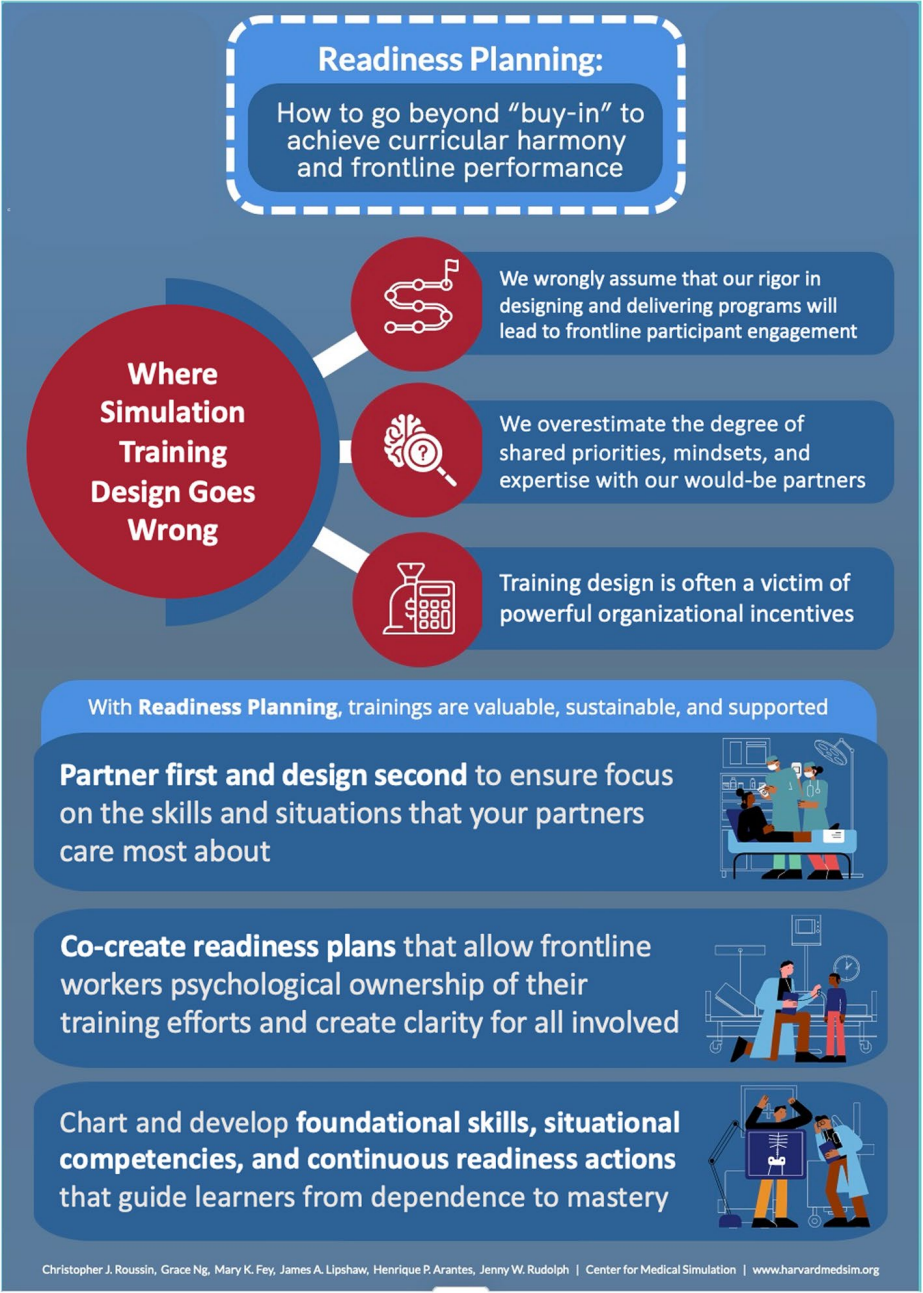

“The readiness is all.” -William Shakespeare, Hamlet

## Main text

To develop learning and training programs that key healthcare partners will value, support, and sustain over time, we simulation leaders face a daunting task. First, today’s healthcare system leaders are at best confused, or at worst cynical, about the practical value of “education” programs of all sorts including simulation-based learning programs. These feelings arise because simulation is often associated with “check-box” educational activities intended to meet various (e.g., accreditation) requirements. This view of simulation-as-education or simulation-as-compliance risks distraction from simulation programs’ potential to help with clinician readiness and team performance issues among other pressing problems.

Second, we simulation program leaders often don’t find ourselves at the table for allocating resources to solve key problems, or don’t consider this part of our role. Yet we have deeply relevant expertise for readying clinicians and teams for myriad practical challenges. We fail to grab the attention of busy health system leaders because we lack a robust narrative to bridge what we offer and what potential partners need. “Education” and “compliance” are well-intended labels but can unintentionally disconnect simulation from “real work.”

Our third problem is this: If we are lucky enough to capture health system leaders’ attention and have resources to tackle a practical problem, we are faced with the question of “how to best shape our programs and demonstrate high-impact change?”. Tackling how to choose, prioritize, and sequence the elements of a training program can seem overwhelming. We often try to cover too many objectives at once, default to a programmatic comfort zone, or end up training on skills extraneous to high-performance front-line care priorities.

### Blind spots in our current design process

How did we get to a place where well-meaning and well-trained simulation leaders can’t consistently partner with health system leaders or unit managers to solve key front-line problems? We argue that it is due to three blind spots in our current design processes.

**We wrongly assume that our rigor in designing and delivering programs will lead to front-line participant engagement and positive impact.** Rigorous application of training design principles is necessary, but not sufficient, to ensure the positive impact of simulation programs on front-line clinical performance. We use sophisticated methods such as backwards design, Kern’s curricular creation steps, and staged learning towards developing efficient and effective programs [1, 2]. However, this rigor is not enough to induce clinical leaders to protect time and space for their staff’s participation. Without deep alignment with clinical partners’ mindsets and priorities, training development efforts inevitably fall short of success [3, 4].

**We overestimate the degree of shared priorities, mindsets, and expertise with our would-be training partners.** Simulation programs and teams are often located away from the clinical frontlines and, despite best intentions, are also culturally distanced from clinical work, priorities, and mindsets. This distance contributes to a tradition of disjointed and off-target training designs. The just-in-time, high-impact trainings created at the onset of COVID-19 worldwide illustrate a massive gap between business-as-usual simulation program development and those programs focused on the glaring readiness needs of the front-line clinical workforce [5–8]. The visibility and urgency of COVID-19 readiness bridged sim-educational and front-line knowledge “silos” and required the two communities to co-own, co-create, test, and run readiness-oriented training quickly [9–12]. These powerful *counter examples* illustrate the necessity of actively sharing priorities and perspectives with each training development effort.

**Training design is often a victim of powerful organizational incentives.** Our third blind spot is that organizational incentives encouraging the appearance of “legitimacy” invisibly work against simulation leaders’ attempts to build practical training programs. Healthcare organizations, like all enterprises, must demonstrate institutional legitimacy to maintain status and to operate legally. They do so by conforming to local, state, and national accreditors’ training requirements [13]. What results is an overemphasis on training focused solely on creating an appearance of compliance and legitimacy. The widespread adoption of such ceremonial training practices in healthcare leads to a “legitimacy façade,” where form supplants function and true learning is compromised. This resource-intensive practice diminishes the perceived value of simulation, demotivates training specialists, and disconnects simulation-based training from its potential impact on real-world work [14, 15].

These blind spots represent a trap for would-be training organizations, as they create negative impressions of the value of training (often referred to as “education”) and present a barrier to the development of future efforts. Managers and front-line workers naturally resent the wasted time and effort associated with misaligned, superficial, or mistargeted training programs. This all creates a downward spiral of perception where each subsequent effort at creating relevant programs is seen with added cynicism or distrust [16]. Simulation team members may adapt to the normality of a low-impact role. Without a useful precedent, front-line clinical leaders can’t imagine a training that is central to the preparation of their workforce.

### The promise of readiness plans

Let’s consider the signs that a specific training program matters to an institution or clinical department: it is funded, staffed, and scheduled. It operates at the rhythm of the team and is not an “add-on.” It is not done on a volunteer basis by participants or instructors and not completed in off hours that infringe on personal or family time.

A crucial difference between readiness planning and conventional training design is that, unlike mandatory check box trainings, the “psychological ownership” of readiness goals—what clinicians will be ready to do—lies primarily *with the trainees* (both leaders and front-line), not the simulation program. The term “psychological ownership” is said to answer the question, “What do I feel is mine?” [17]. Psychological ownership describes my level of investment in something and increases when it is closely tied to my identity, feelings of safety, and my self-efficacy [18]. Understandably, the pop-up trainings to help clinicians be ready for a variety of COVID-related clinical challenges met this criterion [7].

A “boundary object” is a tool that facilitates communication and collaboration [19, 20] across professional groups that may have varying knowledge and interests [21]. Readiness plans serve as powerful boundary objects—they are co-created documents that clarify priorities and facilitate collaboration between simulation team members and representatives from the clinical workforce. Boundary objects powerfully translate concepts (e.g., context, scope, timing) for both communities to enable productive action, as was necessary in the impromptu collaborations between simulation programs and the front-line workforce during the COVID-19 pandemic. In the rest of this article, we outline the distinctive process through which readiness planning unifies training designers, organizational leaders, and front-line workers’ motivation and incentives.

### Outline of this paper

In this paper, we describe:

- How we learned from practice to develop and refine the readiness planning process.
- How to build a readiness plan: It is a “boundary object” that bridges professional silos.
- Three readiness plan use cases.
- Implications, controversies, and considerations related to readiness plans.

### How the need for readiness plans propelled us to develop and refine “use cases”

A “use case” is an illustration and exemplar of how a new process, service, or product might be used [22, 23]. An effective use case, like a strong prototype [23, 24], allows the “end user” to envision how the product or service solves a problem or helps them meet a goal. Well-designed use cases are a proposed solution arrived at through iterative dialogue with goal-oriented stakeholders, customers, or other end-users [25].

We were motivated to invent readiness planning both by our own failures and successes in securing partnership to build simulation-based trainings. Our varied experiences have involved collaborations with hospital, clinical unit and program leaders [26], malpractice insurers [27], quality and safety leaders [28], and simulation leaders around the world. We noticed a pattern associated with our most successful and impactful efforts. Where we partnered to create learning experiences that were valued and enduring, we had constructed a boundary-spanning communication process and a “boundary object” that allowed us to share, translate, and sometimes transform the ways we each thought about a problem [20]. We invented readiness planning to formalize this process and meet our need to be consistent in our successes.

Our mixed training and consulting organization (CR, GN, MF, JL, JR) works to prepare individuals to design, develop, and lead impactful simulation-based training programs and to prepare healthcare organizations and teams to implement sustainable systems of applied learning (including simulation) to support high-quality, positive, and safe healthcare practice.

We co-developed more than 800 readiness planning examples and cases with participants in our simulation leadership and instructor training courses between 2019 and 2023, and through this process, we moved toward an ever more intuitive approach to readiness planning and subsequent training design. We realized that each participant in our course was creating an applied case for their real setting that they could use as a launching pad for collaborative work upon returning home. In our consulting work, we refined the readiness plan format and readiness planning process based on implementation challenges and insights developed with our health system clients and partners.

### Developing a readiness plan

#### The pre-curricular bridge

The language of “readiness” is the magnetic core of a narrative that pulls front-line workers and leaders together with simulation leaders. A readiness plan, in its simplest and best form, lists the things that clinicians and clinical teams must be able to do competently to complete their jobs successfully. The content of readiness plans, specific skill and situational competencies, are the sturdy planks of a “pre-curricular” bridge between front-line doers and training enablers (e.g., simulation experts). Readiness plans use the backwards design principle of beginning with the end in mind [29], but with a twist from the discipline of design thinking: It first asks front-line leaders and training designers to agree on what are the key *situations* students or practicing clinicians must be ready for [3]. This process is a pre-curricular step that uses the most pressing situational priorities as a north star (e.g., managing a violent patient). Readiness plans create a laser focus on training necessities and avoid burdening everyone with extraneous ideas. Readiness plans (1) use priorities defined with stakeholders and partners about gaps and goals in a specific domain [28], and then (2) divide the readiness needs (and associated training designs) into stages that allow health system colleagues to be consistently ready to achieve these key goals or address these key gaps [30, 31].

This list, the readiness plan, becomes the basis for training development. Gerald Grow [30] and Roussin and Weinstock [31] described how learning stages—foundational skills, situational competencies, and learning from real-life situations—can take the learner along a path from dependence to self-directed situational mastery. Every job is composed of situations to be managed. Each situation has a scope, context, and logic of engagement. Within situations, there is a set of interlocking skills that need to be mastered individually before they can be performed in situational context. The front-line team knows its performance context and tells the stories of readiness needs. The simulation team are listeners, guides, and co-developers of the readiness plan and the resulting training designs.

We next present three prototype readiness plans—our use cases. Although these are not actual intact cases representing the exact circumstances in a single hospital, each use case is a composite of settings and readiness plans developed with partners in real contexts.

### Use Case 1: readiness of community hospital team members to manage precipitous deliveries

#### Are we ready to manage an obstetrical patient delivering precipitously outside of the Labor and Delivery (L&D) Unit?

Community Hospital A has been serving members of their surrounding urban and suburban communities for over 150 years. However, in recent years, the area’s population and demographics have shifted dramatically, leading many childbearing families to move to the surrounding mid- and low-cost living areas. As a result, not only did the birth volume double from approximately 500 to 1000 births per year between 2019 and 2023 but the number of high-risk patients and the range of complications experienced by these patients also increased proportionately. 

In the attempt to accommodate the growing patient population, the hospital recruited several new obstetricians and nurse-midwives to join the department, hired additional nursing staff, and even converted several old hospital rooms into L&D rooms. Aggressive training efforts within L&D prepared the new hires to meet the volume demand. However, many patients were unprepared for their impending births and several near misses raised concerns about the hospital’s ability to manage emergency deliveries that originate outside of the L&D unit—e.g., in the Emergency Department or in the hospital parking lot. Staff voiced their worries about readiness to deliver safe and high-quality maternity care to the surrounding population.

#### The readiness planning team

The Women’s Health Service-Line leaders recognized the need for rapid improvements and decided to investigate the causes of issues. Early in the design process, the workgroup recognized that Emergency and Children’s Health Service Lines and the Hospital Security Department are key partners in providing safe, high-quality management of precipitous deliveries outside of L&D, and they were invited to design efforts. This design team found that while staff are knowledgeable within their roles, they are used to managing typical patients in a low-volume setting [3, 4]. Teamwork and communication during labor and delivery emergencies were overall poor. As expected, ER staff not only lacked specialized training and experience to handle the high-stress nature of high-risk obstetrical complications, but also struggled utilizing advanced communication protocols such as Situation-Background-Assessment-Recommendation (SBAR) in requesting support and patient transfers. Security and parking staff were generally unprepared to request help and provide basic immediate support for patients and families. Additionally, the leadership team found that practice standards and protocols were outdated and inconsistent with current best practices. Based on their findings, the service-line leaders enlisted partnership with their internal simulation training team to co-create a training program.

The workgroup identified two priority situations for which staff need to be ready: managing precipitous deliveries outside of L&D and patient transfers. The multi-professional workgroup designed updated practice standards and developed a readiness plan (Table 1) [11, 13–17].

**Table 1:**
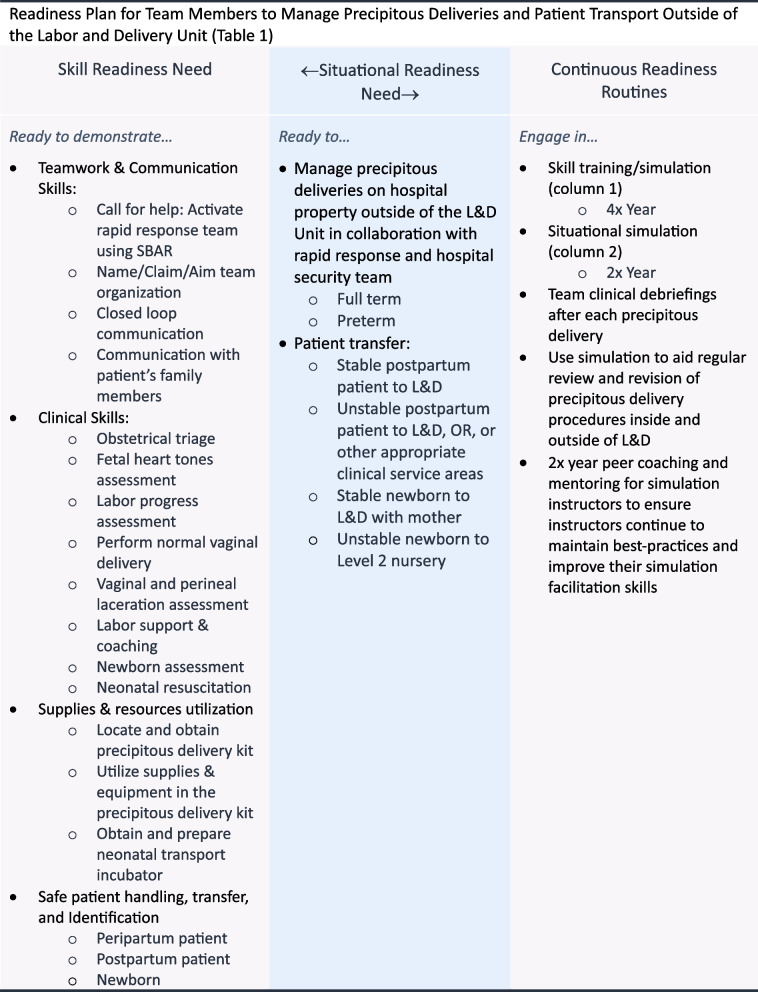
Readiness of team members to manage precipitous deliveries and patient transport outside of the labor and delivery unit (low- to mid-volume semi-rural setting)

Based on this readiness plan, a comprehensive training program was developed, scheduled, and implemented, including simulation-based learning and training using the SimZones model and framework [31]. Leadership supported paid time for all staff to participate in get-ready simulation-based training efforts as well as debriefing and mentoring activities associated with real events. These included skill training (SimZone 1) for sub-teams followed by situational learning and training (SimZone 2) involving multi-disciplinary team members.

### Use Case 2: readiness of team members to recognize and manage violent patients

#### Are we ready to recognize and manage violent behavior from an adult male patient?

A large academic hospital in the Northeastern USA, Hospital B, was facing a crisis regarding the recognition and management of violent patients in multiple patient units. Recent incidents with violent patients had led to injuries among clinicians, mostly nurses, causing a wave of resignations and a significant decline in staff morale. The hospital’s safety and readiness concerns were at an all-time high, as employees felt unprepared to recognize and manage violent patients. An investigation revealed a lack of standardized protocols and training for identifying and managing violent patients—including both proactive and reactive elements. The team took an evidence-informed approach to readiness plan design, utilizing a practical, limited literature review in cooperation with the simulation team. Based on this review, they found that the hospital staff faced challenges commonly identified in the literature [32].

#### The need for situational readiness

Patient de-escalation and restraint episodes varied wildly in approach and outcome. A growing cloud of fear and anxiety around violent patients hung over many patient encounters. Staff reported feeling unequipped to identify early warning signs and de-escalate situations and greatly feared participation in patient restraint episodes. Many were unclear on how to coordinate with the Behavioral Response Team (a set of specially trained clinicians and security officers who can calm or restrain violent patients).

#### The readiness planning team

With leadership support, a partnership-based workgroup of unit-based representatives, safety experts, and training experts began a readiness planning initiative. They researched and integrated evidence-based practice to identify the specific skills and situations staff needed to master to manage violent patients safely and effectively [33, 34]. The workgroup developed a readiness plan (Table 2).

**Table 2:**
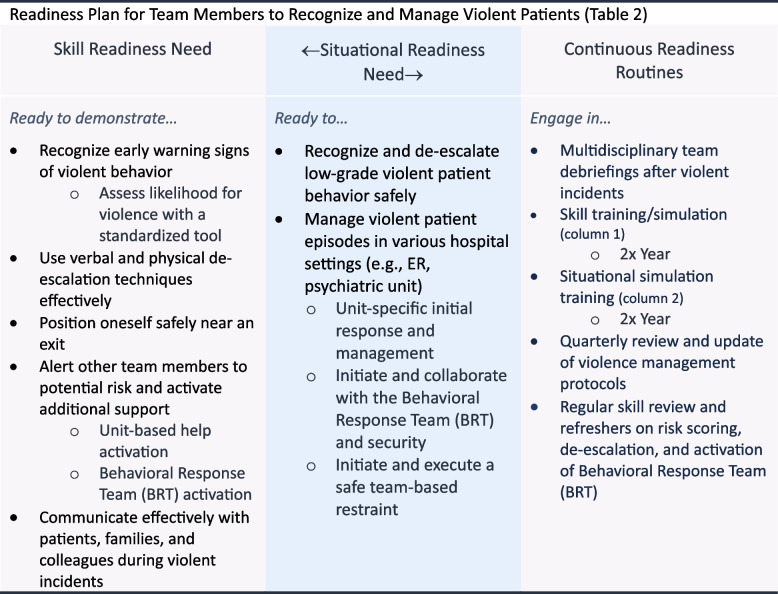
Readiness of team members to recognize and manage violent patients

Based on this readiness plan, a comprehensive training program was developed, including simulation-based learning and training using the SimZones framework. By implementing readiness planning and a SimZones-based training program, the hospital aimed to equip staff with the skills and readiness to manage violent patients, improve staff morale, and create a safer work environment.

### Use Case 3: readiness of new graduate nurses to recognize and manage patient deterioration

#### Are we ready to recognize signs of patient deterioration in a timely manner and escalate care to avoid preventable harm?

Consider a medical-surgical unit in a tertiary care hospital, Hospital C, that had a higher-than-average number of missed or delayed Rapid Response Team calls. In a review of past cases, clinical leaders found that patient vital signs had been declining for a significant period before Rapid Response or Code Team interventions were called, revealing a delay in recognition by nursing staff. On this unit, more than half the staff are registered nurses with less than one year of experience. The unit has a turnover rate exceeding 50% per year, and many of those who left were in their first year of practice. Exit interviews with departing novice nurses revealed common concerns: they felt overwhelmed by the high severity of patient cases, lacked the necessary skills to effectively care for them, and experienced intimidation and lack of support when seeking help from more experienced colleagues. This has led to a demoralizing cycle of high turnover among nursing staff, affecting both the remaining staff and leadership.

#### The readiness planning team

A Clinical Nurse Specialist initiated naming the issue as “failure to rescue” [35] and approached the nurse manager to propose a training program to improve skills in the recognition and initial management of deteriorating patients. The nurse manager initiated the formation of a training advisory group that included RNs, nursing assistants, a respiratory therapist, the Clinical Nurse Specialist, an ICU physician, an applied learning specialist, and an assistant nurse manager. A retrospective analysis of the medical records determined that most patients who required a rapid response or who suffered a cardiac arrest were post-operative patients. The workgroup met, gathered data from across the unit, researched best practices, and developed a readiness plan (Table 3) [35–37].

**Table 3:**
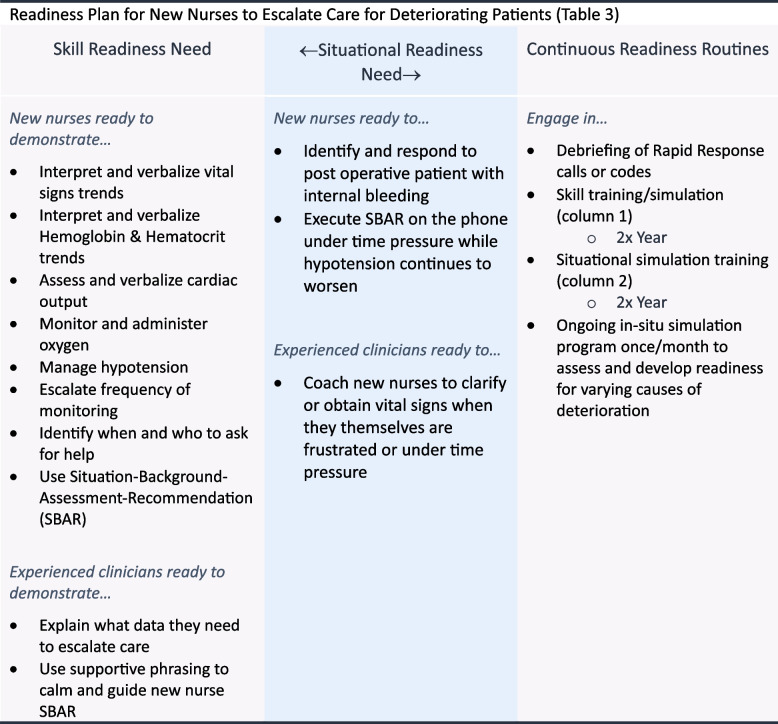
Team readiness to escalate care for deteriorating patients

The readiness plan was used as the basis for training design. Once all RN staff had participated in foundation skill practice in the simulation center, a program of in situ simulations was initiated. In situ simulations took place twice per month on various shifts. After 6 months of in situ simulations, rapid response and cardiac arrest calls dropped by 20%. Based on the success of the simulation program, the simulation advisory committee then recommended a program of routine clinical debriefing to build psychological safety and workflow improvement even further [38].

## Discussion: positioning ourselves as partners

The power of readiness planning is simple—it leads us to the “right” training designs. Just below the surface, it also leads us to a better understanding of our role in creating simulation, nay “readiness,” programs. That is, *we are partners first and designers second*. Put differently, our designs become meaningless unless preceded, and surrounded, by partnerships. We would be better served by viewing ourselves as co-creators of workforce readiness, rather than creators of simulation programs. Traditional training designs fail to clear at least two important hurdles of successful innovation—embedding in the adopters’ network and solving key problems *as defined by the adopters *[4]. In our case, the “adopters” of the innovation of *readiness-focused* simulation are front-line staff, with simulation staff working as expert partners. Our partnering skills must improve, and our organizations must position us to be effective partners across the clinical landscape.

### The power of readiness planning and implications for the role(s) of training designers

Readiness planning also guides us to a fundamental connection between situational competencies and core foundational skills. While skill competencies are the bedrock foundation of competent performance [39], the center of readiness plans are the situations that compose our work. From these situations, we *extract* the foundational skills that we must learn and demonstrate. Readiness planning leads us to avoid two key traps: (1) trying to practice situations (e.g., mock codes, acute situational training) without understanding and building the skills inside those situations, and (2) trying to translate core skills into real-world performance without situational training. The story of readiness planning is clear—to perform in these situations you must first learn the core skills involved, then learn how to combine and apply those skills to the situation. Without each of these pieces, the result is unreadiness. From unreadiness springs discontent, culture-collapse, and unsafe healthcare.

## Conclusion

### Opportunities and barriers to implementation of readiness planning

The key to implementing readiness planning is to recognize that we are partners first, designers second. Psychological ownership of the training’s impact must be shared, and belong primarily, with our partners. This requires us to create a multi-professional design team that includes readiness planning expertise. These efforts are greatly eased when health system executive-level leadership appreciates and supports the approach. Each of the “use cases” featured in this paper illustrates the importance of such a team. In our experience, attempts at readiness planning without representation from essential partners are unlikely to be sustained.

It follows that the most obvious barriers to readiness planning are found in inertia. We are not used to partnering in this way—between clinical groups (aka tribes) and among clinicians and training or simulation experts. Any implementation of readiness planning is a jolting (and very positive!) moment of change, rather than an iteration or tweak. The good news is that healthcare is primed to accept new thinking and approaches, and more open to somewhat radical change, in this moment of widespread cultural challenge.

Readiness Planning is directed toward the core preparation and maintenance of the work team *as defined by the work team and its leaders*, with the first two readiness columns documenting the “get ready” need and the third column addressing the “stay ready and improve readiness” need. The third column can, and should, include simulation-based efforts toward testing and improving process, facilities, and teamwork approaches. Ongoing work in “translational simulation” is focused on rigorously defining applications of simulation that can improve healthcare quality and safety, enhance provider capabilities, and optimize system processes among other benefits [40]. We believe that, as clinical teams become accustomed to documenting readiness needs, the full promise of translational simulation will be realized as teams “pull” rigorous simulation to their needs, in contrast to experiencing simulation as primarily “pushed” toward them through safety and quality organizations and initiatives.

### Future directions for readiness planning

We are freshly amazed every day at the positive effects of using readiness-oriented language in place of education-oriented or compliance-oriented language. “We need our team to be ready for…(performance imperative goes here!)” is an inspiring, and clear, way to start a sentence, or a paragraph, or a conversation…about training for performance. This brings us back to simulation. The potential of simulation to improve performance and patient safety is the “hook” that brought most simulation professionals to their roles. We need to take the next step, moving from a narrative of rigorous design to a narrative of rigorous partnership. The future, and present, direction for readiness planning is to simply do more of it—under any reasonable name or format.

## Data Availability

N/a.

## Abbreviations

L&D: Labor and delivery
SBAR: Situation-Background-Assessment-Recommendation Protocol
